# Comparative Anatomy and Physiology

**Published:** 1886-08

**Authors:** 


Comparative Anatomy and Physiology. *By* F. Jeffrey Bell, m. a.; *Professor of Comparative Anatomy at King's College. 16mo, pp. 555, Illustrated. Philadelphia:* Lea Brothers & Co. *Chicago:* A. C. McClurg & Co.

We have here an interesting treatise, the subjects being introduced in a somewhat original manner. The author first directs attention to the question of living matter, treating it comprehensively as relating to animals and plants, beginning with a definition of protoplasm, and following it up in its develment into tissues and organs. He then, as he claims in his preface, "writes about organs rather than groups of animals," giving details of structure. This will prove to be a most valuable addition to the library of the student who desires to acquaint himself with the fundamental principles concerning the anatomical structure, development and physiology of the organs of lower animals, and for comparing the same parts of one animal with those of another. The writer's style is lucid and terse. It affords one pleasure to peruse the pages of this production. F. C. S.

